# Hot Anchors: A Heuristic Anchors Sampling Method in RCNN-Based Object Detection

**DOI:** 10.3390/s18103415

**Published:** 2018-10-11

**Authors:** Jinpeng Zhang, Jinming Zhang, Shan Yu

**Affiliations:** 1 Brainnetome Center and National Laboratory of Pattern Recognition, Institute of Automation, and Center for Excellence in Brain Science and Intelligence Technology, Chinese Academy of Sciences, Beijing 100190, China; shan.yu@nlpr.ia.ac.cn; 2 University of Chinese Academy of Sciences, Beijing 100049, China; 3 State Key Laboratory for Manufacturing Systems Engineering, Xi’an Jiaotong University, Xi’an 710049, China; zhjmpt@163.com

**Keywords:** image object detection, RCNN, Faster RCNN, Light Head RCNN

## Abstract

In the image object detection task, a huge number of candidate boxes are generated to match with a relatively very small amount of ground-truth boxes, and through this method the learning samples can be created. But in fact the vast majority of the candidate boxes do not contain valid object instances and should be recognized and rejected during the training and evaluation of the network. This leads to extra high computation burden and a serious imbalance problem between object and none-object samples, thereby impeding the algorithm’s performance. Here we propose a new heuristic sampling method to generate candidate boxes for two-stage detection algorithms. It is generally applicable to the current two-stage detection algorithms to improve their detection performance. Experiments on COCO dataset showed that, relative to the baseline model, this new method could significantly increase the detection accuracy and efficiency.

## 1. Introduction

Image object detection is a very classical and foundational computer vision task, which has been studied for many years and still attracts much attention. It is relatively more complicated than the well known image classification task, because the potential target objects on an image not only contain various categories but also have diverse sizes and uncertain locations. The goal of object detection is not only to classify these objects but also to locate them, so it actually contains two sub-tasks, i.e., classification and location. This task is widely used in many fields, such as pedestrian recognition [1], fruit detection [2], music symbol detection [3], visual measurement [4], 3D shape recovery [5], etc. In the early years, this task was mainly based on the manual features and the performance was relatively poor [6]. But in recent years, based on deep neural networks, there were significant developments [7,8,9,10,11]. The current mainstream methods can be grouped into two categories: The region-based methods [6,7,10] and the region-free methods [8,12].

The region-based methods usually adopt a two-stage pipeline. Firstly, a region proposal network classifies the pixel-wise candidate boxes into the foreground and the background, and then the foreground boxes are chosen to create the positive samples, while the background boxes are chosen to create the negative samples. Secondly, a region-wise subnetwork is designed to classify these samples and to refine the candidate boxes. The region proposal network can filter out most of the background samples in the first stage, so the final searching space for the second stage is relatively small, leading to a very good detection performance. The Fast RCNN [13] (RCNN refers to the region convolution neural network) showed a high accuracy on mainstream object detection benchmarks [14,15]. Inspired by this, many other effective methods gradually emerged, including Faster R-CNN [7], R-FCN [16], Mask R-CNN [17], RON [18], etc. However, due to the two-stage design, the computation burden and efficiency of this type of methods are still far from optimal.

The region-free methods directly compute final bounding boxes from each pixel on the CNN-based feature maps by fully convolutional networks (FCNs), and therefore they are also called one-stage method. YOLO [12], SSD [8], and their variants [19,20,21]are the typical examples of this category. Due to ignoring the pre-selection process of the candidate boxes, these methods exhibited a high efficiency, but also brought a new serious foreground-background class imbalance problem. Though adopting hard example mining (HEM) techniques [8,22,23] can suppress the background samples, the detection accuracy is still not very competitive.

Considering the two-stage methods, for example Faster RCNN [7] and Light Head RCNN [10], generating candidate boxes (also called anchors) on the feature maps is an indispensable and very important operation in the overall pipeline. As the detection targets may appear anywhere in the image, the position of candidate boxes are completely unknown. Accordingly, all of these methods adopt uniform sampling strategy, in which all candidate boxes are generated by sampling the pixels on the feature maps at a fixed pixel interval. With this strategy, all the candidate boxes are uniformly distributed on the feature maps, which can be called as uniform anchors.

But are the uniform anchors optimal? Obviously, if we can optimize the operation of anchors sampling, all latter operations in the detection pipeline will benefit from it, and as a result the overall network will be further optimized. In this paper, we will study this question and propose a much better anchor sampling method, referred to as hot anchors, to generate anchors on the CNN-based feature maps. This method is generally applicable to the current two-stage detection algorithms and can be used to improve their performance.

In summary, we make the following contributions in this paper:

- We propose the criteria that can be used to optimize the anchor sampling method in the object detection pipeline. The best sampling method should have the maximal target hit rate as well as the minimum sampling coverage rate.
- We propose an improved anchors sampling method to generate anchors on the feature maps, which is called hot anchors. It is a heuristic sampling method, which makes full use of the CNN-based feature information of images to locate the center pixels of the target objects.
- Experiments on benchmark tasks showed that our method can be incorporated to the current two-stage algorithms to improve their accuracy and efficiency.

## 2. Related Works

Object detection is a very classical task in computer vision. It aims to find out the potential object instances in an image without other auxiliary information. In order to achieve this purpose, one must take full advantage of the image feature information. Therefore, object detection algorithms are naturally rooted in the image feature extraction algorithms.

In the early years, based on the manual image features, such as SIFT [24], SURF [25], HOG [26] and so on, many classical object detection algorithms have been proposed [6,27,28]. These algorithms use manual-designed feature descriptors to represent an object, and then sweep through the entire image with a sliding-window to find those regions with a class-specific maximum response. The DPM [6] and its variants [27,28] are the typical examples of this type of method and have been the dominant methods for years.

Recently, the deep convolutional neural networks (CNNs) gradually dominate the image feature extraction, accompanied by the emergence of many successful detection algorithms based on CNNs. For example, R-CNN [29] and its variants usually combine a region proposal method (such as Selective Search [30], EdgeBoxes [31], MCG [32], etc.), and a post-classification method (such as SVM). These methods exhibited a significant improvement on the detection accuracy. After that, the SPP-Net [33] and Fast R-CNN [13] respectively proposed Spatial-Pyramid Pooling method and RoI-Pooling method that allow the head-subnet to reuse features computed on the CNN feature maps. Furthermore, the Faster R-CNN [7] suggested a region proposal network (rpn) to achieve even better accuracy and efficiency. With this pipeline, there are many other methods proposed, which are all called the two-stage methods. Among them, R-FCN [16] tried to reduce the computation time with position-sensitive score maps. FPN [34] built a feature pyramid through combining the multi-scale CNN feature maps, which can greatly improve the detection accuracy on small objects. RON [18] proposed a reverse connection method to detect on multi-scale feature maps of CNNs and proposed an objectness prior method to reduce the search space of objects. Mask R-CNN [17] proposed RoIAlign method and creatively added a mask prediction branch to the network, which can fulfill three different tasks in one inference. Light Head R-CNN [10] proposed a thin feature map and a much smaller subnetwork to gain a significant improvement in both the accuracy and efficiency. However, these two-stage methods must filter a large amount of anchors by a region proposal network during the training and inference, which result in high computation burden and time cost.

Meanwhile, other methods, such as YOLO [12] and SSD [8], provided one-stage solutions, and they have the advantage of efficiency but the disadvantage of relatively low accuracy. They all face a serious issue of sample imbalance [6,8,35,36], which causes two problems [9]: (1) Training is inefficient as most locations are easy negatives that contribute no useful learning signal; (2) as a whole, the easy negatives can overwhelm the training and lead to degenerate models. A common solution is to perform some form of hard negative mining [8,22,23] that samples hard examples during the training or more complex sampling/reweighing schemes [23]. Recently, RetinaNet [9] exhibited a significant improvement in the accuracy. It used a novel focal loss function to focus the training on a sparse set of hard examples and prevented the vast number of easy negatives from overwhelming the detector during the training process. But the issue of sample imbalance is still a hindrance for these methods to gain further improvement in their performance.

Taken together, the one-stage methods have the limitation of low accuracy caused by the imbalanced sampling, while the two-stage methods suffer from the problem of low efficiency due to too much anchors sampled on the feature maps. Here we propose a new heuristic sampling method, called hot anchors, to generate relatively fewer but high quality anchors on the feature maps, which can improve the current well known two-stage algorithms in both the accuracy and efficiency.

In addition, different from the earlier Selective Search [30] method and Edge boxes [31] method, which use the lower-level features of the original image to locate the potential objects, our method reuse the deep CNN features as heuristic information to generate anchors on the multi-scale feature maps and can be naturally integrated in the network pipeline for end-to-end training and inference. Our method is also different from the anchors sampling method used by YOLOv2 [19], YOLOv3 [20] and T-CNN [37], in which they focus on how to optimize the scales and ratios of anchors, but we focus on how to optimize the locations of anchors.

## 3. Our Method: Hot Anchors

### 3.1. Detection Input Space

In image object detection, the potential target objects on an image will constitute the input of the algorithm. But these target objects may have an arbitrary size and ratio, and moreover may appear at any location of the image. This means that the input space of the detection algorithm is completely unknown. As a result, one has to firstly estimate the input space and generate a lot of candidate boxes as the estimate values of the target objects.

The candidate boxes, also called anchors, can be characterized by three parameters: Location, scale, and ratio. Location represents the center coordinate of the object ground-truth box on an image, while scale and ratio represent the object size and shape, respectively. All the combinations of the possible values of location, scale and ratio will commonly constitute the detection input space, and the algorithm should select the proper ones from this input space. The input space can be represented in Equation (1).

(1) InputSpace=Anchor(location,scale,ratio)

In fact, the scale and ratio can be two continuous variables, so the number of possible combinations are infinite, thereby leading to the infinite InputSpace. In actual computation, we usually set scale and ratio as some fixed discrete values. They are usually considered as the hyper parameters of the algorithms, which can be manually set. For example, a popular setting is that scale is [128, 256, 512] and ratio is [1/2, 1, 2]. The algorithm YOLOv2 [19] provided a data driven method to analyse the scale and ratio of anchors, in which they adopt k-means clustering on the training set bounding boxes to automatically find good priors(anchors), and then reduce the number of anchors from 9 to 5 but still keep a good performance. In fact, the location, scale and ratio are independent with each other, and they constitute the three different optimization dimensions for anchors, and in this paper we only choose the location to study. In addition, the total number of the resulting candidate boxes will be the product of the number of different values of the three parameters. In this situation, the size of the InputSpace (the number of candidate boxes) will be mainly determined by the anchor locations. Taking an image of 1024×1024×3 as an example, the number of total locations (pixels) can be as large as one million, but the center pixels of target objects only occupy a very small proportion of them.

### 3.2. Anchors Sampling

Obviously, we must sample form the original input space to reduce the number of candidate boxes and therefore reduce the search space of the detection algorithm. Considering the scale and ratio are usually quantified to fixed values and can be treated as hyper parameters, we only need to examine the location parameter. So, our analysis will mainly focus on how to efficiently sample the locations on an image to accurately locate the target objects.

Currently, the well known two-stage algorithms all adopt a common method to sample anchors: Scanning one or several of the multi-scale CNN-based feature maps at a fixed pixel interval to generate anchors with different scales and ratios. This method is referred as the uniform sampling, in which the final anchors will uniformly distribute in the feature map plane. The locations of anchors are only constrained by the shape of the feature map and the fixed pixel interval. The fixed pixel interval, which is just the sampling stride, can help to control the density of anchors. Because the size ratios between the original image and its CNN feature maps are fixed values for a given CNN model, the anchors sampled on the feature maps can be converted to the original image panel through multiplying them by the size ratios. This means that the uniform sampling on the CNN feature maps is equivalent to the uniform sampling on the original image. Accordingly, the underlying assumption is that each pixel on the image has the equal probability to locate the centers of target objects.

Is the uniform anchors the best strategy? Compared with the very few target objects on an image, most of the anchors are redundant and invalid, and these too many anchors will increase computation burden and influence the detection accuracy. Clearly, more efficient method to sample anchors is highly desirable.

In the training process of the two-stage detection pipeline, we need to compute the intersection-over-union (IoU) between the anchors and the ground-truth boxes to judge whether an anchor can be a good match to any ground-truth box. Because the anchor scales and aspect ratios are set as the fixed values for all the anchor locations, and the aspect ratios are symmetrically designed (such as [1/2, 1, 2]), the matching process started with choosing two boxes with similar scale and ratio by a predefined IoU threshold. Under such circumstance, the decrease of the center distance usually (but not always) leads to the increase of IoU. Though the relationship between the center distance and IoU is not strictly monotonic, once the center distance reaches the minimum (zero), the IoU will reach its maximum regardless of the scales and ratios of two boxes. Therefore, we firstly try to sample the anchors with the center distance as small as possible. After the anchor locations have been optimized, the quality of the anchors will depend only on the scale and aspect ratio, which will be another independent optimization problem. On the other hand, we usually match one ground-truth box with many anchors, so considering these anchors as a whole, they actually spread around the center of the ground-truth box. Therefore, the smaller average center distance usually means a denser distribution of anchors, and further hints a larger average IoU for all these anchors. So, based on the above considerations, we firstly use the center distance between anchors and ground-truth boxes to examine the quality of anchors. This is an essential analysis on the location parameter of anchors without the influence of the scale and ratio.

As good anchors usually fall in the neighborhood of a ground truth box, we need to further define a distance threshold to judge whether an anchor is an appropriate one. This is equivalent to defining an IoU threshold for two boxes to judge wether they can be matched with each other for the training. Specifically, we define a valid hit if the center distance between a ground-truth box and any anchor is equal to or smaller than a distance threshold $d_{0}$; an invalid hit if the center distances between a ground-truth box and all the anchors are larger than the distance threshold $d_{0}$. This can be expressed in the following Equation (2). The ground-truth box is denoted as gt_box for simplicity, and the gt_box_hit represents whether a ground-truth box has been hit by any anchor. *d* represents the center distance between a ground-truth box and its nearest anchor, and $d_{0}$ is the distance threshold. A result of 1 represents the ground-truth box has been hit by at least one anchor, while 0 represents the ground-truth box has not been hit by any anchor. The center distance is the Euclidean distance between the ground-truth box center and the anchor center.

(2) $$gt\_box\_hit=\left\{\begin{array}{cc} 1 & d\leq d_{0}\\ 0 & d>d_{0} \end{array}\right.$$

For example, when the distance threshold $d_{0}$ is 5 and the center distance between a ground-truth box and its nearest anchor is 3, the gt_box_hit of this ground-truth box will be 1; while if their center distance is 6, the gt_box_hit of this ground-truth box will be 0. The first situation means that this ground-truth box has been validly hit by an anchor at a distance of 3. The second situation means that this ground-truth box has not been hit by any anchor under the condition of threshold $d_{0}=5$.

Given an image, we firstly define the sampling coverage rate as the ratio between the number of sampling pixels and the number of total pixels, and then define the target hit ratio as the ratio between the number of valid hit target pixels and the number of total target pixels. Target pixels refer to the center pixels of the ground-truth boxes on an image. This can be expressed in Equations (3) and (5), in which an image has a total of $N_{ap}$ pixels and a total of $N_{tp}$ target pixels (that is $N_{tp}$ ground-truth boxes). The number of sampling pixels is $N_{sp}$ and the number of valid hit pixels is $N_{hp}$. The valid hit refers that the Euclidean distance between a target pixel (the center of a ground-truth box) and a sampling pixel (the center of an anchor) is less than the distance threshold $d_{0}$. The number of valid hit pixels $N_{hp}$ can be calculated by summing the gt_box_hit across all the ground-truth boxes of an image, as shown in Equation (4).

(3) $$coverage\_rate=\frac{N_{sp}}{N_{ap}}$$

(4) $$N_{hp}=\sum_{i=1}^{N_{tp}}\left(gt\_box\_hit_{i}\right)$$

(5) $$hit\_rate=\frac{N_{hp}}{N_{tp}}=\frac{\sum_{i=1}^{N_{tp}}\left(gt\_box\_hit_{i}\right)}{N_{tp}}$$

If we sample all the pixels of an image, it is certain that we can hit every target pixel on the image. This is just the uniform sampling with the highest density. In this situation, the sampling coverage rate is 100% and the target hit ratio is also 100%. This is the simplest but not the optimal sampling method. **The optimal sampling method should hit the most target pixels with the minimum sampling pixels**. In other words, we must consider the sampling efficiency and treat the anchor sampling as an optimization problem, which is missing in the current two-stage methods using the uniform sampling.

### 3.3. Hot Anchors and Hot Feature Map

**Figure 1:**
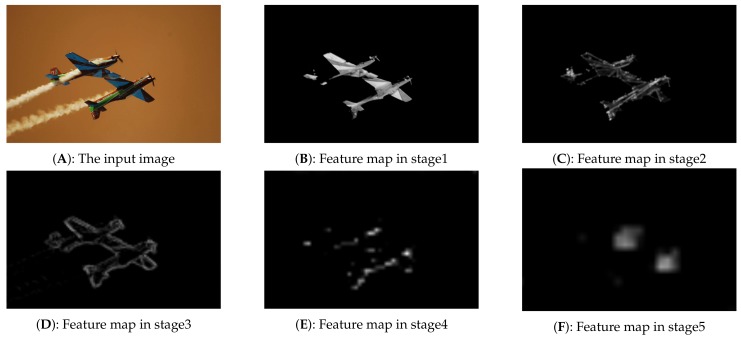
The multi-scale feature maps generated by ResNet50 [38]. (**A**) is the original input image with a size of 1024×1024×3. The (**B**–**F**) respectively come from the five different stages of the multi-scale convolutional neural network (CNN) feature maps and their panel size are 512×512, 256×256, 128×128, 64×64, 32×32. In this figure, all of them has been resized to the same size for convenient display.

The uniform anchors actually only make use of the image shape information, but ignore the image feature information. When an image passes through the convolutional neural networks, multi-scale feature maps will be generated. The pixel values of these feature maps are also valuable information for anchor sampling. Figure 1 shows an image in COCO2014 dataset [15] and its multi-scale feature maps generated by ResNet50 [38], in which we can clearly observe the evolutionary process of the target objects. Obviously, the pixel values are very informative for anchor sampling. Specifically, the objects usually occupy the regions with high activation values, while the background pixels usually occupy the regions with lower activation values. Based on this observation, we propose a heuristic sampling method to generate anchors on the feature maps, which is called Hot Anchors. Hot, a word drawn from the color-coded temperature map, means that the heuristic information we used is the pixel activation values of the CNN feature maps, and it also hints that the pixels with high activation value have a higher probability to locate a target object.

Because the feature maps generated by CNN are multi-scale and each stage has different sizes, some preprocessing steps are necessary. Considering the anchor sampling should be low computational cost, the preprocessing method cannot be too complex. Assuming the feature map size is donated as [batch,channel,height,width], we directly sum a feature map along its channel axis to generate a new feature map, which is referred as the hot feature map. Hot means that we expect this feature map can faithfully reflect the high activation region in feature space, so that it can be used to sample hot anchors. The hot feature map has the same height and width with the original feature map, but its channel dimension is one, so its size can be donated as [batch,1,height,width]. batch is the number of the input images for each calculation of CNN. We can compute the hot feature map on each stage of the multi-scale CNN feature maps, and eventually obtain the multi-scale hot feature maps.

We still need to give a threshold to judge which pixel on the hot feature map should be chosen as an anchor location. Because the hot feature map of each image is different, so we use the mean pixel value of the hot feature map as a threshold to make an adaptive judgment for each image. If a pixel value is greater than the threshold, it will be treated as a positive hot anchor. But this may lead to different numbers of hot anchors on different images, so in order to make sure the number of hot anchors on different images is the same and is convenient for program processing, we multiply the mean pixel value by a coefficient *k* as the final threshold. *k* can be initialized to 1. If the number of hot anchors is less than the presupposed value, multiplying *k* by 0.8 and try to resampling until the number of anchors is enough. Contrastingly, if the number of hot anchors is more than the predefined value, we randomly eliminate the redundant ones. This can be expressed as the following Equations (6) and (7), in which the hot feature map is donated as HFM, the channel of the original feature map is donated as *C*, and the mean pixel value of the hot feature map is donated as mpixel, with (x,y) representing the pixel coordinates on HFM.

(6) $$HFM=\sum_{c=0}^{C}feature\_map\left(batch,c,height,width\right)$$

(7) HotAnchors=Anchor((x,y),scale,ratio)ifHFM(x,y)≥k×mpixel

If we only want to sample anchors on one of the multi-scale CNN feature maps, we can select the middle stage among them. On the one hand, the top stage is too abstract and often has a very small size, so small objects may be ignored and the total number of pixels may also be too few to sample enough anchors. On the other hand, the bottom stage is not abstract enough and may has too many background details. The middle stage, which usually compromises between the image details information and the object semantic information, can be a good choice. However, this is just a general guiding principle, and the actual situation should be determined according to the backbone network used for feature extraction. For example, if we use the ResNet50 or ResNet101 [38] as the backbone network, there will be a total of 6 stages and the stage-3 or stage-4 can be considered as the middle stages.

If it is desirable to sample anchors on several stages of the CNN feature maps, we can execute the same sampling operation on each of the stage separately, and then integrate the multi-scale results together as the final sampling results. For some feature fusion architecture, such as FPN [34], the hot feature maps can be created based on the amalgamated pyramid feature maps. Taken together, the hot feature maps can be created on any form of multi-scale CNN feature maps.

### 3.4. Hit Distance Histogram

In the anchor sampling operation, each sampling pixel corresponds to multiple pairs of scale and ratio. In other words, each sampling pixel has several boxes bound to it. Therefore, each ground-truth box will have several nearest anchors, which means that the center distances between the ground-truth box and these anchors are the minimum among all anchors. Because the scales and ratios are the same fixed values for all anchors, so these neighbor anchors usually contain the anchor that has the maximum IoU with the ground-truth box. The neighbor anchor with the maximum IoU will be the best matched anchor of a ground-truth box, and their center distance (donated as $d_{hit}$) can be called the hit distance of the ground-truth box. It means that this ground-truth box will be hit by the anchor at a distance of $d_{hit}$. Each ground-truth box has its best matched anchor and, correspondingly, its hit distance $d_{hit}$.

Obviously, the hit distances of all the ground-truth boxes on an image will not be same, and they are distributed within an interval. In principle, we expect all of these hit distances to be as smaller as possible, so that each ground-truth box can get a higher quality best matched anchor. So, in order to investigate the anchors sampling quality more comprehensively, we further study the shape of distribution of the hit distances of all ground-truth boxes on the images in a dataset.

**Figure 2:**
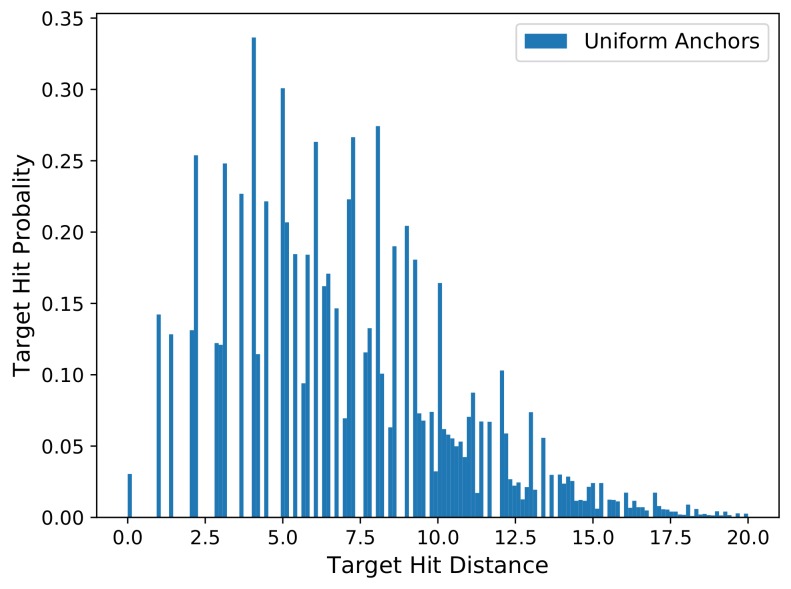
The hit distance histogram calculated in COCO2014 mini-validate set. All anchor locations are uniformly sampled on the original images with a sampling stride of 14 pixels. The size of the original image is 1024×1024×3. We expect the histogram distribution can be as closer to *Y*-axis as possible.

When we go through all the images in a dataset, we can sample anchors on each image, and calculate the best matched anchors for all the ground-truth boxes on these images, and then compute their corresponding hit distances. Finally, we can calculate a distribution histogram of the hit distances for all the ground-truth boxes in the dataset, which is referred as the ***hit distance histogram***. Figure 2 shows a hit distance histogram calculated from the mini-validation set of COCO2014 dataset, which contains about 4952 images and about 36,800 ground-truth boxes. The horizontal *x*-axis of the hit distance histogram is the distance bins, which start form zero. The vertical *y*-axis is the normalized quantity ratio of the ground-truth boxes that fall in each distance bin. When the number of images and ground-truth boxes are very large, the quantity ratio of the *y*-axis can be considered to be the target hit probability, which reflects the probability that a ground-truth box can be hit by any anchor at any distance.

## 4. Experiments and Analysis

We use three anchor sampling methods to do contrast experiments. We will firstly make an analysis on the target hit rate to examine whether our method is valid and efficient. Then we will take a more comprehensive investigation on the anchors quality by comparing their hit distance histogram. Finally, we will incorporate our method to the current two-stage object detection algorithms to examine whether it can improve their performance.

The first method is called uniform anchors, in which all anchors are uniformly sampled on the original image at a fixed sampling stride. The sampling stride can help to adjust the anchors density, with larger sampling stride corresponding to fewer anchors. In many detection algorithms used currently, the uniform anchors are usually sampled on the CNN feature maps at first, and then all of them will be normalized and converted to the image domain. So, we directly use the uniform sampling with different stride on the original image to mimic the uniform sampling on the feature maps. They are essentially equivalent.

The second method is called edge anchors, which is a kind of heuristic sampling on the original image. This method directly uses the canny edges of the original image as heuristic information to sample anchors. The underlying rationale is that any object must have its edges, so it is natural to directly sample anchors on the pixels containing object edges. Firstly, we calculate the canny edges of an image and obtain a binary edges image, which has the same height and width with the original image. Then we randomly sample a certain amount of none-zero pixels on the binary edges image as the final anchors.

The third method is the hot anchors we proposed, which uses the CNN features of the original image as the heuristic information to guide the sampling operation.

### 4.1. Hit Rate Analysis

We use the COCO2014 [15] dataset as our benchmark and its training set has a total of about 110 k (1 k = 1000) images and about 860 k ground-truth boxes, which is large enough to perform in-depth analysis. We resize the original image to 1024×1024×3 and keep the shape of the image content area unchanged, so the total number of pixels is 1024×1024 and the anchors will be sampled from these pixels. The uniform anchors will be sampled on the original image with a fixed pixel interval, and the edge anchors will be sampled on the canny edge image of the original image, while the hot anchors will be sampled on the multi-scale feature maps generated by a pre-trained convolution network (ConvNet). The ConvNet backbone we adopted is ResNet101 [38], whose feature maps have five stages with corresponding strides of [2,4,8,16,32]. So, the plane size of the feature map at each stage will be [512×512,256×256,128×128,64×64,32×32].

**Figure 3:**
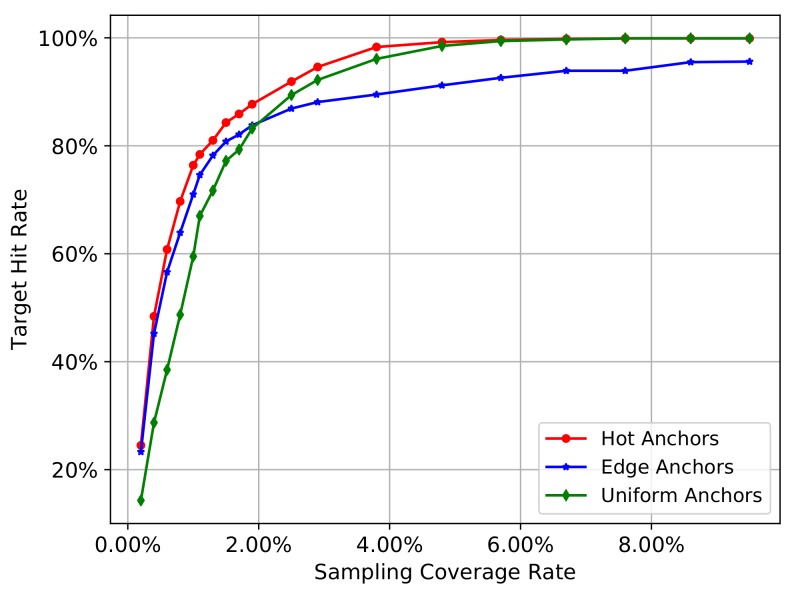
The target hit rate curves of three sampling methods. Hit distance threshold $d_{0}$ is 5.

**Figure 4:**
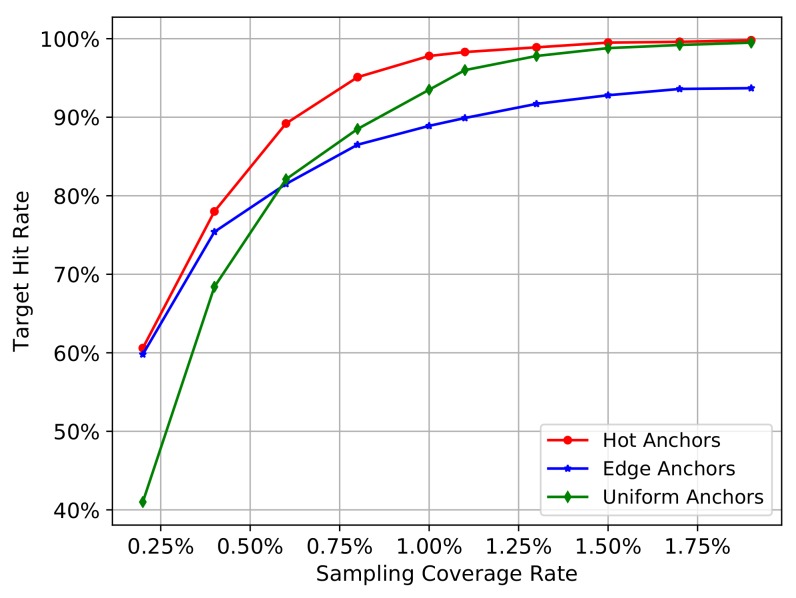
The hit rate curves of three sampling methods. Hit distance threshold $d_{0}$ is 10.

We will go through all the images in COCO2014 training set to calculate the mean hit rate by Equation (5) on all images. We gradually increase the number of sampling pixels, so the sampling coverage rate will also gradually increase. In this process, we can investigate the change trend of the mean hit rate of different sampling methods. In addition, the distance threshold $d_{0}$ will influence the calculation of gt_box_hit in Equation (2), and therefore influence the result of target hit rate in Equation (5), so we set the distance threshold $d_{0}$ as 5 and 10 to carry out the experiment separately. The Figure 3 and Figure 4 show the experiment results.

In the Figure 3 and Figure 4, the red curve represents the hot anchors, blue curve represents the edge anchors, and the green curve represents the uniform anchors. In the Figure 3, when the number of sampling pixels increases from 1000 to 100,000, the sampling coverage rate increases from nearly 0.10% to nearly 10.00%, and the mean hit rate on COCO2014-training-set also gradually increases from nearly 20% to nearly 100%. In the Figure 4, when the number of sampling pixels increases from 2000 to 20,000, the sampling coverage rate increases from nearly 0.20% to nearly 2.00% , and the mean hit rate on COCO2014-training-set also gradually increases from nearly 40% and 60% to nearly 100%. Comparing the Figure 3 with Figure 4, we can see that the bigger the hit distance threshold $d_{0}$ is, the smaller the sampling coverage rate will be given the same target hit rate.

From the two figures mentioned above, we can obtain the following results. Firstly, the target hit rate of the three sampling methods gradually increases with the increasing sampling coverage rate, so the sampling coverage rate is the most critical factor that affects the target hit rate.

Secondly, we can see that the green curve reflects a relatively low hit rate at the beginning stage, while the blue and red curves reflect a relatively high hit rate. This means that the heuristic sampling really has a big advantage over uniform sampling when the sampling coverage rate is relatively low. The heuristic information, including the canny edges on the original images and the activation values on CNN feature maps, indeed contributes to sample the pixels closer to the target objects. In other words, the pixels with much more heuristic information will have a greater probability to locate a target object, demonstrating the validity of the heuristic information.

Thirdly, when the coverage rate raises up to a critical value, the uniform anchors (green curve) will outperform the edge anchors (blue curve). This reflects the shortcomings of heuristic sampling methods. Because the edges of different objects are not the same, the big objects usually have much more edges than the small objects. In other words, the heuristic information contained in different objects is not equivalent, and their distribution on the image is usually imbalanced. So if the edges overly accumulate on a few larger objects, the sampling pixels will also accumulate on these objects and therefore be regional imbalanced. Especially when the image contains many large objects and small objects at the same time, the small objects have less heuristic information and may be passively ignored in the condition of finite sampling pixels. On the contrary, the uniform anchors don’t have this problem, and their distribution on the image is completely regional balanced. Moreover, when the number of the sampling pixels gradually increases, the sampling stride of the uniform anchors will become smaller and smaller, accompanied by the increasing density of anchors. Finally, the uniform anchors will gradually exhibit advantages over the edge anchors with the increasing sampling coverage rate.

Fourthly, the hot anchors method exhibits the advantages of both uniform sampling and heuristic sampling. Considering the pooling operation of ConvNet, the sliding-window with a fixed-size in pooling operation can be equivalent to the uniform grids on the original image, and each pixel of the pooled feature map will come from these uniform grids. In other words, if we convert the pixels on the feature map to the original image, the activation values will distribute among these uniform grids on the original image. Consequently, the heuristic sampling based on the activation values is actually controlled by these uniform grids. These uniform grids can be thought as another form of uniform sampling, which will contribute to restraining the regional imbalance of the heuristic sampling. Thus, the hot anchors method is actually a heuristic sampling controlled by uniform grids, making it a hybrid method, enjoying the functional advantages of both approaches. Therefore, we can see the curve of hot anchors is always the best one.

Taken together, both the uniform and heuristic sampling have its advantages and disadvantages. The former can restrain the regional imbalance but ignore the useful image feature information, while the latter utilize the image feature information but the imbalanced distribution of heuristic information may lead to ignoring the small objects with less heuristic information. The hot anchors make full advantage of characteristics of CNN feature maps and provide a good compromise between these two aspects, leading to much better performance compared to the uniform and heuristic sampling methods.

The results (Figure 3 and Figure 4) show that the edge anchors exhibit poorer performance compared to the hot anchors over the entire tested range of sampling coverage rate, and it only works better than the uniform anchors when the sampling coverage rate is very small. Therefore the edge anchors method is clearly inferior compared to the other two methods. Thus to simplifying further analyses in the following experiments, we focus on the comparison between the hot anchors and the uniform anchors.

### 4.2. Hit Distance Histogram Analysis

In this experiment, we analyze the distribution characteristics of hit distance histogram of the two sampling methods with better performance, uniform anchors and hot anchors. We set the number of sampling pixels to 5 k, 10 k, 30 k, and 50 k respectively to calculate the distance histogram. We still use the COCO2014 training set as the benchmark.

**Figure 5:**
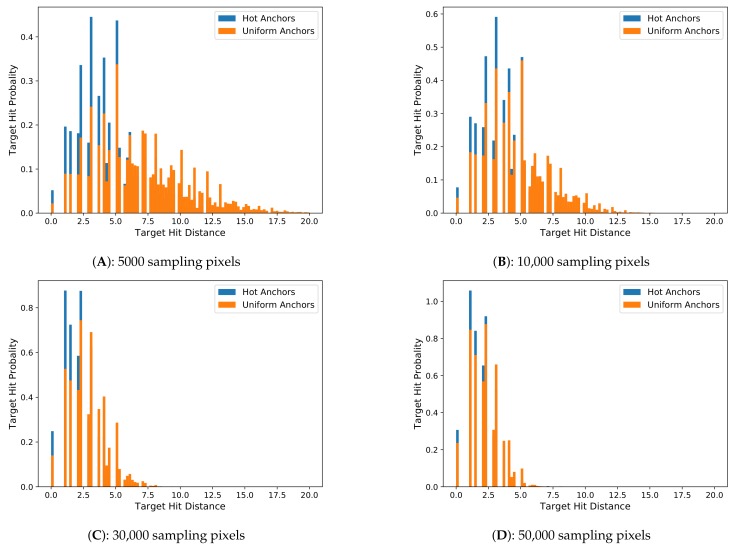
The hit distance histogram with different number of sampling pixels. The figures (**A**–**D**) have 5000, 10,000, 30,000, and 50,000 sampling pixels, respectively. The distribution of hot anchors is always closer to *Y*-axis than the uniform anchors when the number of sampling pixels is the same.

In the Figure 5 we can see that when the number of sampling pixels are the same, the uniform anchors have a wider range of distribution and a larger mean distance value, while the hot anchors have a relatively narrower range of distribution and a relatively smaller mean hit distance. In addition, with the increase of the sampling pixels, the histograms of the two methods all gradually move to the left, closer to the *Y*-axis. These results illustrate that the number of sampling pixels is a key factor determining the quality of anchors, but importantly, with the same sampling pixels, the hot anchors always exhibit better results, demonstrating a higher sampling efficiency.

The calculation of the hit distance histogram covers all of the 860 k ground-truth boxes contained in about 110 k images in COCO2014 training set. The results obtained from such a large dataset provide a comprehensive picture regarding the quality of anchors in different methods. The ground-truth boxes and their corresponding matched anchors will constitute the learning samples to carry out a supervised learning process, and the learning goal is to regress these estimated anchors to their corresponding ground-truth boxes. So it is conceivable that if the anchors have a bigger area overlap with their corresponding ground-truth boxes, the regression learning will be much more easy and accurate. Under the situation of fixed scales and ratios, the location will be the only parameter to adjust the area overlap between the ground-truth boxes and the anchors, so a good anchor sampling method should make all anchors as closer to their corresponding ground-truth boxes as possible. Based on this consideration, the closer the distribution of hit distance histogram to the *Y*-axis, the smaller the deviations between the anchors and their corresponding ground-truth boxes will be, and therefore it will be easier for the anchors regression in network training and inference. From the Figure 5, we can see that the hit distance histogram of hot anchors obviously has a better performance than the current used uniform anchors, providing a better choice for the two-stage detection algorithms.

### 4.3. Recall-to-IoU Analysis

Just as Equation (1) shows, the scale and ratio are also the key factors for anchors, so it is necessary to analyze the final performance of hot anchors with these two parameters. In general, the scales and ratios are manually set as hyper parameters, and we set the scales to [512, 256, 128] and the ratios to [1/2, 1, 2], respectively. Under this setting, the number of anchors on each sampling location will be 9.

**Figure 6:**
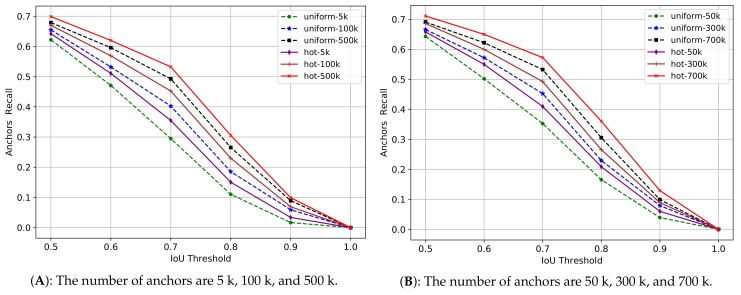
The recall of anchors with different IoU threshold. 1 k equals 1000. uniform refers to the uniform anchors and hot refers to the hot anchors. The number of sampling pixels is 1/9 of the number of anchors. We show the results in two separate figures (**A**,**B**) for clear observation in case the curves are too dense.

**Figure 7:**
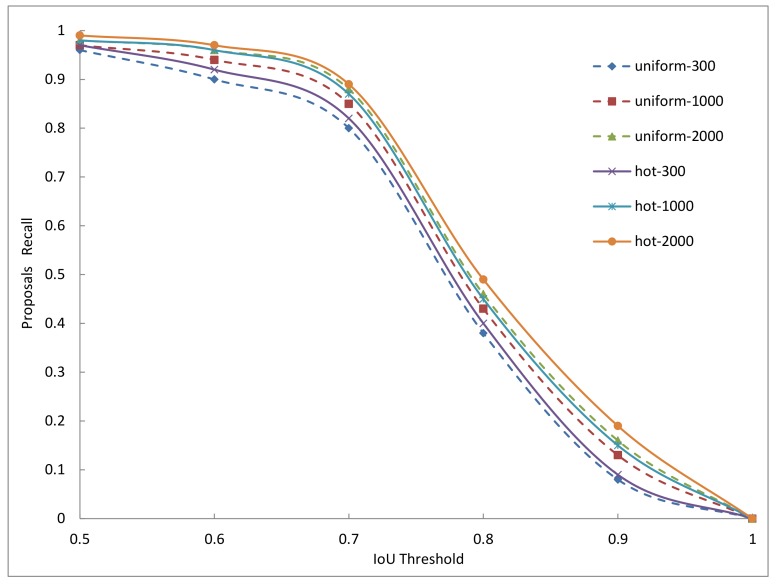
The recall of proposals with different IoU threshold. uniform refers to the uniform anchors and hot refers to the hot anchors. For example, the uniform-300 means the top-300 proposals computed from the uniform anchors.

In the two-stage methods, the anchors will be firstly processed by rpn (region proposal network [7]) to generate proposals, and then these proposals will be used to crop RoIs from the feature maps, and at last these RoIs will be used for object classification and positioning. So the quality of anchors will influence the quality of proposals, and further influence the quality of RoIs, and finally influence the detection performance. In general, we use the intersection over union (IoU) to measure the area overlap between two boxes, and a higher IoU value with a ground-truth box means a better quality for an anchor or for a proposal. In other words, for both anchors and proposals, we expect that they can perfectly cover the input space constituted by the target objects on an image. This coverage ability can be measured by the recall of anchors or the recall of proposals. Given an IoU threshold, the recall of anchors or the recall of proposals can be calculated by the ratio between the number of qualified ones (judged by the IoU) and the total number of ground-truth boxes on an image. By going through all the images in a dataset, we can calculate the recall curves with different IoU thresholds. We use the COCO2014 minival set containing about 5000 images to do the experiments, and the results are shown in Figure 6 and Figure 7.

From the above two figures, we can see that with the IoU increasing from 0.5 to 1, the recall of anchors quickly decreases from about 0.7 to 0 for both uniform anchors and hot anchors. With the same IoU threshold, when the number of anchors increases, the two methods outperform each other alternately, which explains that the number of anchors is always the key factor to the recall of anchors, and therefore, is the key factor to the quality of anchors. But with the same IoU threshold and the same number of anchors, the hot anchors always have a higher recall value than the uniform anchors.

The Figure 7 reflects the quality of proposals generated by rpn in the trained Faster-RCNN models on COCO2014 dataset, in which two anchors sampling methods are used. The figure shows the result of 300, 1000, and 2000 proposals. The N proposals are the top-N ranked ones based on the confidence generated by these methods. In the figure, we can see that with the same IoU threshold and the same number of proposals, the recall of proposals computed from the hot anchors has a relatively higher performance than the proposals computed from the uniform anchors. But with the number of proposals increases, the two methods outperform each other alternatively, indicating that the number of proposals is very important to their quality. On the whole, from the Figure 6 and Figure 7, we can see that the hot anchors are a better choice for rpn to generate relatively high quality proposals.

### 4.4. Detection Algorithms Analysis

At the end, we will apply the hot anchors sampling method to two typical two-stage algorithms to test its performance, which are Faster RCNN [7] and Light Head RCNN [10]. In the above analyses, we have taken the activation values of the CNN feature maps as the heuristic information to judge which pixel has the larger probability to locate the target objects on an image. The anchors based on these pixels will form the positive samples, and in addition, we need to select the negative samples at the same time.

The negative samples refer to the background regions on an image, which don‘t contain valid object instances. Because the ConvNets feature extractor essentially plays a role of suppressing the image background information and strengthening the foreground object information, so the object semantic information on the feature maps will be more pronounced at the deeper layer of the network. This also means that the activation values on background regions will be lower than the foreground regions. So, we can again use the activation values as the heuristic information to locate the pixels belonging to the background regions. Similar to what we do for the positive anchors, we will set another threshold to select the pixels with low activation values as the negative anchors. The value of this threshold can be calculated through multiplying by another coefficient k′ to the mean activation value of the hot feature map. If a pixel activation value is smaller than the threshold, it will be treated as a negative anchor location. We initialize $k^{\prime }$ to 0.4 in the following experiments. Thus, through this method, the locations of both the positive and negative anchors can be obtained at the same time by a higher positive threshold and a lower negative threshold, respectively. Then, we can generate anchors with different scales and ratios on these locations. Because there are several anchors on each pixel location, we need to further apply the intersection-over-union (IoU) to filter the anchors. Specifically, if the IoU is greater than 0.7, the anchor will be a positive anchor, while if the IoU is less than 0.5, the anchor will be a negative one.

In these experiments, we limit the number of anchors on each image to several fixed values (5 k, 20 k, 50 k), and then compare their performance on benchmark datasets. We change the number of anchors only by adjusting the number of locations, but keeping the scales and ratios to be fixed. For Faster-RCNN, we set the anchor scales as [128, 256, 512], and set the anchor ratios as [1/2, 1, 2]. For Light Head RCNN, we set the anchors scales as [32, 64, 128, 256, 512], and set the anchor ratios as [1/2, 1, 2]. For both of them, before feeding the proposals generated by rpn (region proposal network [7]) into RoIs prediction subnetwork, we need to apply the non-maximum suppression (NMS) to filter some excessive overlapping proposals, so we set the intersection-over-union (IoU) threshold for NMS as 0.7.

**Figure 8:**
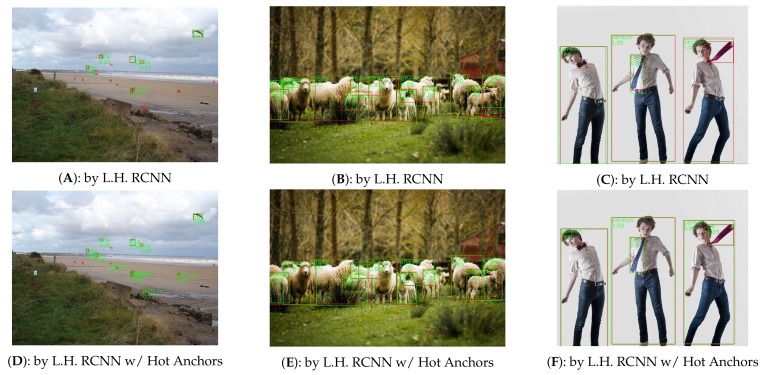
The comparison of detection results between Light Head RCNN [10] and Light Head RCNN with Hot Anchos. The (**A**–**C**) are the results detected by Light Head RCNN (Abbreviated as L.H. RCNN), and the (**D**–**F**) are the results detected by Light Head RCNN with Hot Anchors . The green boxes tagged by class label and classification score are the detection results, while the red boxes are the ground-truth boxes. All the images come from COCO2014-minival-set and have been resized to the same size for convenient display.

**Figure 9:**
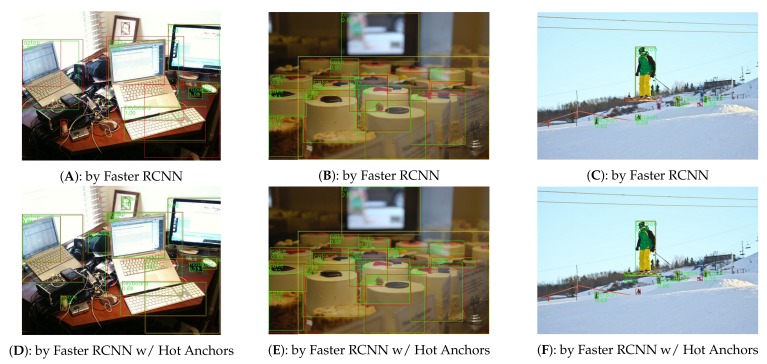
The comparison of detection results between Faster RCNN [7] and Faster RCNN with Hot Anchos. The (**A**–**C**) are the results detected by Faster RCNN, and the (**D**–**F**) are the results detected by Faster RCNN with Hot Anchors . The green boxes tagged by class label and classification score are the detection results, while the red boxes are the ground-truth boxes. All the images come from COCO2014-minival-set and have been resized to the same size for convenient display.

The experiments are carried out on COCO2014 [15] dataset, which has 80 object categories, 80 k images in the training set, and 40 k images in the validation set. The 40 k images in the validation set will be further split into 35 k val-minusmini set and 5 k mini-validation set. Following the general setting, we combine the train set and the val-minusmini set to obtain the 115 k images for training and use the 5 k mini-validation images for validation. The optimizer is synchronized SGD (Stochastic Gradient Descent) with a moment of 0.09 and a weight decay of 0.0002. For Faster RCNN, the learning rate is initialized as 0.001 and then is decayed by 0.1× for every 5 epoches (one epoch covers all the 115,000 training images). For Light Head RCNN, the learning rate is set to 0.01 for the first 1.5 M iterations, and 0.001 for later 1 M iterations (one iteration covers one batch of data). The experiments are carried out on the platform with 4 NVIDIA GTX1080Ti GPUs, CUDA 8.0, and cuDNN v6, and each mini-batch has 2 images per GPU. Figure 8 and Figure 9 show some of the detection results.

The Table 1 and Table 2 show the experiment results, in which the numbers with plus sign in the brackets are the difference value of the two methods under the same number of sampling pixels. The “Feature Pyramid” refers to the FPN [34] architecture. The mAP [15] refers to the mean average precision, and the fps refers to the frames per second. We can see when the number of anchors is relatively small, the hot anchors perform much better. This can also be demonstrated by the Figure 3 and Figure 4, in which the hot anchors have a much higher hit rate and shorter distance between the best matched anchors and the ground-truth boxes, and therefore can facilitate the later process of anchors regression. Compared to the uniform anchors, the fewer the anchors are, the more pronounced advantage of hot anchors will have in locating the potential target objects. When the number of anchors is sufficiently large, the accuracy gap (5.3, 4.1, 3.0 *for Faster RCNN;* 3.9, 2.6, 0.9 *for Light Head RCNN*) between these two methods will become very small. This is because the sampling coverage rate is now large enough to lead to very dense anchors, and therefore the effect of the heuristic information is relatively weakened. Thus, the advantages of the hot anchors will be most valuable in the demanding situations where only a small number of anchors can be generated.

In addition, hot anchors also improve the inference speed. Because we can use two different strategies in network training stage and inference stage for hot anchors. In the training stage, the hot anchors contain both positive anchors and negative anchors at the same time, but in the inference stage, the hot anchors only contain the positive anchors. With the help of the activation value of the CNN feature maps, we can make a prejudgment on pixels and only select the pixels with high activation value to generate anchors for the following computation in the network inference stage. All other anchors with relatively lower activation value will not participate in the inference computation, significantly reducing the computation burden. Due to the fact that the number of these low activation anchors is actually very large, such reduction is quite significant. This is an important different compared to the uniform sampling method, in which the anchors on each pixel are all treated indifferently with equal importance, and will be all passed to the following inference computation. In summary, compared with the uniform sampling, the heuristic sampling make full use of the image feature information to reduce invalid sampling and therefore can lower the calculation burden of the subsequent operations in the detection pipeline.

## 5. Conclusions

In this paper, we propose a new, efficient sampling method to generate candidate boxes on the feature maps for detection algorithms, which is called hot anchors. In this method, we use the activation values of the CNN feature maps as the heuristic information to sample the pixels that have a higher probability to locate a target object. This is different with the current mainstream methods, which only use the image size information to do uniform sampling on the image panel. Experiments on COCO benchmark have shown that our method is superior in terms of both the inference accuracy and efficiency. Our method can be incorporated to the widely used two-stage approaches to improve their performance. As the candidate boxes are also used in some one-stage algorithms, we expect that the hot anchors method could also benefit these algorithms in the future.

## Figures and Tables

**Table 1:** The Inference Accuracy in Faster Region Convolutional Neural Network (RCNN) and Light Head RCNN. 1 K equals to 1000.

Methods	Feature Pyramid	Input Size	Inference Accuracy/mAP@[0.5:0.95]					
			Uniform Anchors			Hot Anchors		
			5 K	20 K	50 K	5 K	20 K	50 K
Faster RCNN	×	600×1000	10.5	16.5	30.3	15.8 (+5.3)	20.6 (+4.1)	**33.3 (+3.0)**
Light-Head RCNN	*√*	800×1200	15.2	30.6	41.1	19.1 (+3.9)	33.2 (+2.6)	**41.7 (+0.6)**

**Table 2:** The Inference Efficiency in Faster RCNN and Light Head RCNN. 1 K equals to 1000.

Methods	Feature Pyramid	Input Size	Inference Efficiency/fps					
			Uniform Anchors			Hot Anchors		
			5 K	20 K	50 K	5 K	20 K	50 K
Faster RCNN	×	600×1000	2.9	1.9	1.1	3.2 (+0.3)	2.5 (+0.6)	**2.0 (+0.9)**
Light-Head RCNN	*√*	800×1200	14	11	9	15 (+1)	13 (+2)	**12 (+3)**

## Abbreviations

The following abbreviations are used in this manuscript:

CNN	Convolutional Neural Network
RCNN	Region Convolutional Neural Network
ConvNet	Convolutional Network
HFM	Hot Feature Map
SIFT	Scale Invariant Feature Transorm
NMS	Non-Maximum Suppression
SGD	Stochastic Gradient Descent
